# Unusual Case of Polypus in the Throat and Cavities of the Face

**Published:** 1819-10

**Authors:** 


FOR THE LONDON MEDICAL AND PHYSICAL JOURNAL.

### *Unusual Case of Polypus in the Throat and Cavities of the Face; successfully treated by* Dr. TROWBRIDGE.

ON the 11th of July, 1818, I was requested to examine Samuel B. Turner, aged 16 years, who was brought by his father, to receive, if possible, surgical aid and assistance.

This young man had been, three years previous, attacked with pain in the left eye, extending at times to the fore-part of the head, with difficulty of breathing through the left nostril, and hoarseness.

Nothing was discovered in either nostril, which induced physicians, who first saw him, to suspect the affection to proceed from a polypus.

The second year increased the above symptoms, with the addition of a sensation of fullness in the throat, with occasional bleedings into the mouth, and falling-down of small fleshy substances of the size of filberts.

It was supposed to be a case of *scrofula* by physicians who saw him, and had been treated accordingly! The last twelve months had increased the affection materially; and, to an observing and intelligent mind, must have placed the nature of the complaint beyond all doubt or controversy.

At the time I examined him he was much emaciated: deglutition was so difficult, that he could pass nothing but liquid food, and that required much exertion. The neck was enlarged externally; a large hard substance, of a dark crimson colour, filled the throat and cavity of the mouth, pressing the velum palati to the teeth of the upper-jaw, and resting on the tongue, epiglottis, and uvula, impeding respiration, and rendering it similar to, and as difficult as in, cases of croup. The face, on the left side, was distorted. The eye, pressed forward from its socket, was enlarged; its vessels turgid, and its coats inflamed, with loss of vision; its lachrymal duct obstructed, and its humours running on to the cheek. The face, on this side, was much enlarged. The upper teeth loose, and partly pressed from their sockets. The voice changed; its resonance and tone entirely lost. A tumor appeared externally near the connexion of the temporal bone with the antrum maxillare as large as a common-sized egg, occasioned by a hard substance that had filled the antrum, crowded the bones asunder, and passed out under a portion of the temporal muscle. The patient was affected with a stupor and dozing at times. Pulse frequent, and feeble. On enquiring, found the young man had enjoyed good health previous to this attack of local disease.

Cases of polypi in the nose and throat had frequently been presented to me, and the use of forceps had been made with success in all cases which had fallen under my care. But this case exhibited a different aspect from any I had ever seen or treated. In the patient was exhibited a picture of human distress and misery beyond description. Frequent hæmorrhages from the mouth had reduced him to extreme feebleness, and his situation was like one suffocating in the last stage of croup. The tumor that appeared in the mouth was hard and painful to the touch; when touched or pressed, it occasioned a hæmorrhage, pain in the eye and fore-part of the head. Distressed days and nights, with constant danger of suffocation, attended the poor sufferer.

No case in the whole round of surgical business could be presented, that could excite more interest or exertion in a surgeon to relieve than this.

Extirpation of the tumor from the throat, was the first and principal relief to be given. The danger of fatal hæmorrhage forbid the use of forceps: I had recourse to the ligature. I procured a flexible silver wire, nearly two feet in length, bent it in the centre, and brought the two ends together. My patient was placed in a chair, his head thrown back, and supported by assistance. The bent end of the wire was now introduced into the left nostril, and pushed on till it appeared on the same side of the tumor in the mouth: with the forceps I now brought the wire out of the mouth, opened it at the bent end, and formed a loop sufficiently large to pass on to the three first fingers of my right hand. My fingers, with the wire, were now passed into the mouth, and directed to the fauces, or lower part of the tumor. The exertions of the patient to resist suffocation raised the tumor, so that I passed the noose off my fingers around the depending portion; with my left hand hold of the wire at the nostril, I drew them sufficiently tight to be convinced that the wire was around the tumor. I now with my right hand, with a curved probe, split at the end sufficient to receive the wire, enlarged the noose, and pushed it high upon the tumor and nearly to its base; drawing at the same time firmly the ligature with my left hand. I now passed the wire at the nostril through a silver tube, six inches long, and curved at its extremity sufficiently to pass easily into the posterior nostril. This I pushed deep; and, drawing the ligature firm, fastened it to the ring of the tube.

In passing the ligature around the tumor, it occasioned a violent hæmorrhage: drawing it through the uvula, produced extreme pain in the affected eye and fore-part of the head.

The patient was directed to keep in an erect posture; the ligature to be tightened every day, till inflammation, suppuration, and a detachment of the tumor, should take place.

He passed the first forty-eight hours with but little alteration in symptoms or situation. On the morning of the third day the tied portion of the tumor began to enlarge, and all the complaints it previously caused were much exasperated. Deglutition and respiration were so much impeded, that prompt relief became necessary.

With a scalpel, I incised the portion of tumor which lay in the mouth: it discharged a quantity of grumous blood, which lessened its circumference, and caused the depending part in the throat to stop respiration entirely. I immediately passed a long dissecting-hook, and fastened it as low upon the tumor as possible, and pressed it up and back, which gave the patient an opportunity to breathe. Extraction became immediately necessary. My assistant, Dr. Davis, fastened another hook into the tumor: with the two, I, with much exertion, brought the bottom of the tumor up and out of the mouth, seized it with a pair of forceps, and brought out a large portion. Dr. Davis at the same time made a division as high in the mouth as possible. My patient was now relieved by the removal of nine ounces of the tumor, and his throat cleared, so that he took food of any kind, and respired with ease. A portion yet remained inclosed in the ligature, which remained firm. I directed my patient to take wine and nourishment, and wait the effects of the ligature: this came away on the third day after, and the remaining portion of tumor thrown out of the mouth. My attention was next turned to the tumor in the left side of the face: after convalescing one week, my patient was so much recovered from his feeble state, that I ventured to remove this tumor.

A portion of it had passed out of the antrum, and rested in the muscles and integuments over the alveolar circle.

I first made an attempt to extract it from the antrum and upper part of the face, by making an incision in the mouth through these muscles, or to the projecting portion of the tumor. After exposing it, I placed a firm pair of forceps, and made strong exertions, to no effect: the pain produced was excessive. I abandoned this course, and commenced an incision externally over the tumor near the temporal bone, carried it down over the os maxillare superior, till I came into the incision I had made in the mouth. This exposed to view the two projecting portions from the antrum. I now applied a trephine, and opened into the antrum, and divided the remaining portion of the os maxillare. After dividing some membranous adhesions, I drew out with the forceps the whole tumor, and exposed its base, which was near the termination of the antrum into the posterior nostril.

I now twisted the tumor till it separated from its strong hold. A large artery was divided near this point, and was secured with a ligature. The passage of the antrum into the nostril was sufficiently large to admit the little finger of my right hand, which was passed through into the throat: by this means I ascertained that the whole tumor in the nostril, throat, and face, was removed.

*Remarks.*—This case corresponds with some described by Pott; viz. it was confined to one side of the face, was hard, shed nothing but blood; was painful to the touch, of a dark-purple colour, filled with arteries.

He says further, that such cases ought not to be meddled with. The danger of irritation produced by extirpation occasions the most disagreeable consequences. Mr. Pott describes one other kind of polypi, viz. of a greyish-light colour, sometimes appearing like a membrane filled with water, situated in one or both nostrils, never painful or tender to the touch; can be extracted with no hazard, and but little pain. John Bell, Richter, and other writers, view the subject in a different light; and deny the fact, that polypi are different in their nature, and justify extraction in all cases and stages.

To those who are investigating the subject of polypi, or who are called to relieve those unfortunate persons who may be afflicted with one, the history of Turner's case may be of some use.

---

FOR THE LONDON MEDICAL AND PHYSICAL JOURNAL.

### *Observations on the Treatment of Hysteria.* By J. MAHON, M.D.

